# Objective structured clinical examination to teach competency in planetary health care and management – a prospective observational study

**DOI:** 10.1186/s12909-024-05274-9

**Published:** 2024-03-19

**Authors:** Ulf Teichgräber, Maja Ingwersen, Max-Johann Sturm, Jan Giesecke, Manuel Allwang, Ida Herzog, Frederike von Gierke, Paul Schellong, Matthias Kolleg, Kathleen Lange, Daniel Wünsch, Katrin Gugel, Anne Wünsch, Janine Zöllkau, Inga Petruschke, Kristin Häseler-Ouart, Bianca Besteher, Swetlana Philipp, Urte Mille, Dominique Ouart, Jana Jünger, Jena Planetary Health Collaboration Group, Thomas Kamradt, Mathias Pletz, Andreas Stallmach, Sina M. Coldewey, Ekkehard Schleußner, Ulrich Wedding, Martin Walter

**Affiliations:** 1 Office of the Dean, Jena University Hospital, Friedrich-Schiller-University Jena, Jena, Germany; 2 Department of Radiology, Department of Diagnostic and Interventional Radiology, Jena University Hospital, Friedrich-Schiller-University Jena, Am Klinikum 1, 07747 Jena, Germany; 3 Student Representatives, Jena University Hospital, Friedrich-Schiller-University Jena, Jena, Germany; 4 KLUG-Deutsche Allianz Für Klimawandel Und Gesundheit E.V., Berlin, Germany; 5 Institute of Infection Medicine and Hospital Hygiene, Jena University Hospital, Friedrich-Schiller-University Jena, Jena, Germany; 6 Department of Internal Medicine IV, Jena University Hospital, Friedrich-Schiller-University Jena, Jena, Germany; 7 Clinic of Anaesthesiology and Intensive Care Medicine, Jena University Hospital, Friedrich-Schiller-University Jena, Jena, Germany; 8 Department of Obstetrics, Jena University Hospital, Friedrich-Schiller-University Jena, Jena, Germany; 9 Institute of General Practice and Family Medicine, Jena University Hospital, Friedrich-Schiller-University Jena, Jena, Germany; 10 Department of Internal Medicine II, Jena University Hospital, Friedrich-Schiller-University Jena, Jena, Germany; 11 Department of Psychiatry and Psychotherapy, Jena University Hospital, Friedrich-Schiller-University Jena, Jena, Germany; 12 Department of Psychosocial Medicine, Psychotherapy, and Psychooncology, Jena University Hospital, Friedrich-Schiller-University Jena, Jena, Germany; 13 SkillsLab Jena, Jena University Hospital, Friedrich-Schiller-University Jena, Jena, Germany; 14 Institute for Communication and Assessment Research, Heidelberg, Germany; 15 Program of Master of Medical Education (MME), Medical Faculty Heidelberg, Heidelberg University, Heidelberg, Germany

**Keywords:** Climate change, Communication, Curriculum, Feedback, Objective structured clinical examination, Planetary health

## Abstract

**Background:**

Health professionals are increasingly called upon and willing to engage in planetary health care and management. However, so far, this topic is rarely covered in medical curricula. As the need for professional communication is particularly high in this subject area, this study aimed to evaluate whether the objective structured clinical examination (OSCE) could be used as an accompanying teaching tool.

**Methods:**

During the winter semester 2022/2023, 20 third- and fifth-year medical students voluntarily participated in a self-directed online course, three workshops, and a formal eight-station OSCE on planetary health care and management. Each examinee was also charged alternatingly as a shadower with the role of providing feedback. Experienced examiners rated students’ performance using a scoring system supported by tablet computers. Examiners and shadowers provided timely feedback on candidates` performance in the OSCE. Immediately after the OSCE, students were asked about their experience using a nine-point Likert-scale survey and a videotaped group interview. Quantitative analysis included the presentation of the proportional distribution of student responses to the survey and of box plots showing percentages of maximum scores for the OSCE performance. The student group interview was analyzed qualitatively.

**Results:**

Depending on the sub-theme, 60% -100% of students rated the subject of planetary health as likely to be useful in their professional lives. Similar proportions (57%-100%) were in favour of integrating planetary health into required courses. Students perceived learning success from OSCE experience and feedback as higher compared to that from online courses and workshops. Even shadowers learned from observation and feedback discussions. Examiners assessed students’ OSCE performance at a median of 80% (interquartile range: 83%-77%) of the maximum score.

**Conclusions:**

OSCE can be used as an accompanying teaching tool for advanced students on the topic of planetary health care and management. It supports learning outcomes, particularly in terms of communication skills to sensitise and empower dialogue partners, and to initiate adaptation steps at the level of individual patients and local communities.

**Supplementary Information:**

The online version contains supplementary material available at 10.1186/s12909-024-05274-9.

## Introduction

Physician engagement is needed to provide planetary health care and to initiate and accompany environmental transformation processes at the community level [1]. In addition, health professionals should contribute to reducing the health care footprint of greenhouse gas emissions [2].

At the same time, physicians are concerned about the health impacts of climate change and see themselves as having the responsibility and capacity to facilitate changes at the individual and local community levels [3]. However, few medical schools worldwide have integrated planetary health into their curricula [4]. Learning about planetary health is particularly relevant in terms of application. This means that communication and argumentation play an important role. Management of adaptation steps in clinical prevention and population health is required. In general terms, it is mainly about sensitising, persuading, motivating, and empowering others to act, i.e. to change their behaviour and attitudes. Currently proposed learning formats for planetary health education include lectures, small group courses, use of standardized patients, and rotations through clinics/offices. However, the focus is put largely on developing awareness and knowledge and on integrating content into existing medical curricula without overloading [5–8].

Objective structured clinical examinations (OSCEs) are characterised by a controlled and structured assessment of a wide range of clinical skills and knowledge. A large amount of feedback is possible [9]. Previous studies have shown that students perceive OSCE that includes specific and constructive feedback to be a well-suited learning tool [10–13]. The purpose of this study was therefore to assess the suitability of OSCE as an additional learning format for planetary health care and management.

## Methods

### Study design

In the winter semester 2022/2023, medical students in their third or fifth year voluntarily participated the elective course “Planetary Health Care and Management”, which was offered for the first time at our university. The course consisted of a 20-h online course with virtual teaching, three two-hour workshops, and finally, a two-hour voluntary OSCE. In order to evaluate the suitability of the OSCE for teaching planetary health care and management, a student survey and a student focus group interview were conducted immediately after the OSCE. In addition, students’ subject knowledge and communication skills during the OSCE were assessed. Informed consent to participate in the study was obtained from all subjects.

### Opportunities to gain skills

#### Online course

The online course was provided by OPEN vhb (Bavarian Virtual University, Bamberg, Germany) with content provided by the Universities of Munich, Regensburg, Augsburg, and Würzburg (https://open.vhb.org/blocks/ildmetaselect/detailpage.php?id=295&lang=en, accessed on 19 September 2023). The intended learning objectives (ILOs) of the course included knowledge and critical reflection on the links between climate change and multiple health impacts, classification of the altered dynamics of disease vectors, discussion of nutritional concepts, knowledge of prevention and treatment options, identification of adaptation and transformation needs, and the ability to initiate and shape such processes.

#### Workshops

The workshops were led by committed students and covered the three themes of (1) planetary health: in particular, how to explain the links between the global climate crisis and individual health (co-benefits); (2) the doctor-patient dialogue: in particular, considering the subtopics of physical activity, nutrition, mental health, and obstetric care; and (3) collegial and interprofessional discussion on prescribing metered dose inhalers (MDI), the management of nursing homes during heat waves, the public management of vector-borne diseases, and the use of specific inhalational anaesthetics. The learning objectives were based on the German National Competency-based Learning Objectives Catalogue in Medicine (NKLM 2.0, sections VIII. 2. – 5.; https://nklm.de/zend/menu, accessed on 28 September 2023) and the optional addendum on planetary and global health (https://nklm.de/zend/objective/list/orderBy/@objectivePosition/studiengang/Themen/zeitsemeszei/Themen%20und%20Fachkataloge/fachsemester/Planetare%20und%20Globale%20GesundhGes%20(Stand%201.%20Juli%2021), accessed on 7 October 2023).

#### OSCE

Finally, a competency assessment course was offered, consisting of eight stations for simulating situations on unrelated subtopics of planetary health, with a time frame of 5 min each. The dialogue partners were represented by actors. The content was jointly developed by the heads of nine departments and members of the academic staff of the university hospital, the academic dean, the examination board, and committed students in common agreement. The idea to develop an OSCE on planetary health care and management and to integrate the OSCE into medical teaching came from a postgraduate course (Master of Medical Education, MME) at the University of Heidelberg in the summer semester of 2022. During the course, participants were given the task of developing an OSCE with the involvement of student volunteers.

The planetary health OSCE should focus primarily on the acquisition and training of competencies and secondarily be used for formal assessment. Formal assessment should be process-oriented to provide supportive feedback to learners and to promote improvements in learning and academic teaching. Feedback to examinees was provided by examiners (eight different experienced lecturers who were involved in the design and implementation of each OSCE station), and by a peer, the so-called shadower, who underwent the OSCE as an examinee immediately before or after shadowing the peer examinee. In short, each examinee was also shadowed in turn. The shadower provided feedback on the performance of the assigned peer in all the OSCE stations.

The Stations were (1) advice on physical activity to an overweight 70-year-old patient including information on health-climate co-benefits (general practice), (2) discussion with an employee about changing medical prescribing practice: replacing MDI with dry powder inhaler (general practice), (3) dietary advice to a 35-year-old patient including information on health-climate co-benefits (general practice), (4) environmental risk assessment for mental health of a depressed patient (psychiatric day hospital), (5) advice to a nursing care expert on measures to be taken during heat waves (nursing home), (6) medical advice to a pregnant woman on the risk of preterm birth due to heat stress and preventive measures (gynaecologist), (7) consultation with a local mayor on the Asian tiger mosquito (risk of transmission of vector-borne diseases caused by arboviruses) and preventive measures through public management (public health officer), (8) collegial discussion on replacing of the inhalation anaesthetic desflurane with sevoflurane to reduce greenhouse gas emissions (anaesthesiology).

### Evaluation

#### Assessment of student performance

Examiners who were lecturers in the subject area of the respective OSCE sub-topic, assessed the students’ performance. The assessment system was based on pre-defined tasks to be completed by the examinee. Scoring was supported by the use of checklists on tablet computers at the same time as or immediately after each examination. The maximum score for student performance was a total of 100 points for each OSCE station, including both subject knowledge (including anamnesis, environmental impact/health co-benefits, and options for action where appropriate) and communication skills. Proportion of the total score for the respective components were at the discretion of the lecturers who designed the OSCE stations (Supplemental Table 1).

#### Student survey

**Fig. 1:**
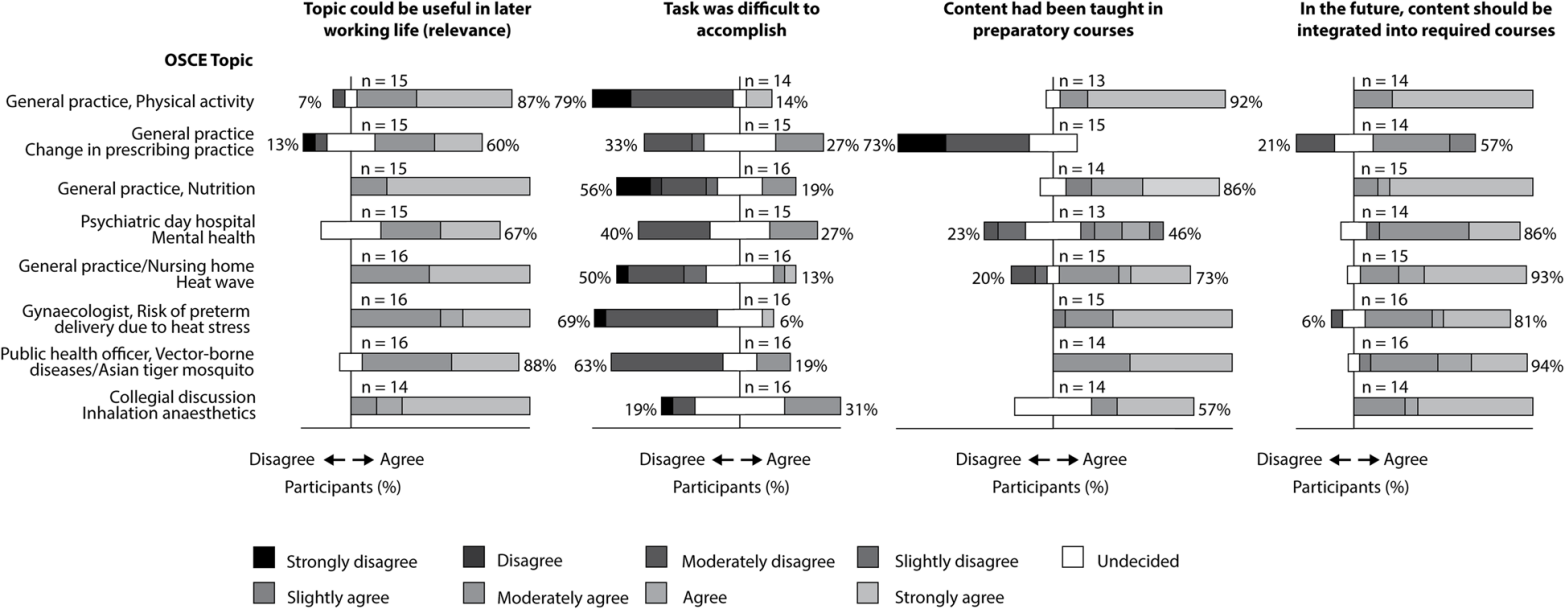
Student survey on voluntary participation in an objective structured clinical examination on eight selected planetary health topics. The OSCE followed a free elective on planetary health that included online courses and in-person seminars. The survey consisted of nine-point Likert scales with four statements for each topic. The figure shows the percentage of respondents who agree or disagree, respectively, and specifies the level of agreement or disagreement. OSCE, objective structured clinical examination

The survey consisted of paper-based, nine-point Likert scales for four statements at each OSCE station (Fig. 1). The scales ranged from strongly disagree to strongly agree with an option for “undecided”.

#### Student focus group interview

Students were asked to participate in a focus group interview about the OSCE they had just experienced. The interview consisted of four open-ended questions posed by a moderator (Tables 1 and 2). Answers were discussed with peers and notes were sticked on a blackboard. The interview was also videotaped.

**Table 1:** Content-related focus group interview with students who underwent the objective structured clinical examination on planetary health

How do you think planetary health should be integrated into teaching?	
**Proposal**	**Reason/Additional comments**
Adopt as a compulsory subject	• Increasing relevance • There will be little additional burden on students • Concise information is preferred to excessive detail • The elective may be considered as an additional course offered to students
Integrate planetary health into existing courses	• Planetary health should not be drowned out as a minor issue • New and existing learning content can be coherent • High learning effect is expected
Address advanced semesters	• Planetary health includes cross-curricular issues that require broad knowledge • Up to date knowledge at the start of the career
Convey knowledge in seminars	• Allows students to actively contribute, discuss, and interact • Allows training of communication skills • Seminars can be varied, inspiring and fun
Use OSCE as teaching and feedback technique rather than as final exam	• Students rated OSCE as more effective than workshops and much more effective than online courses • OSCE puts pressure on the students and thus increases the learning effect • OSCE should be conducted only after participation in seminars and courses • Mandatory examination could have a negative impact on intrinsic engagement
What task/role do you see for yourself in planetary health as a future doctor?	
**Task/Role**	**Reason/Additional comments**
Ensure environmentally friendly use of energy, medicines, and materials in their area of responsibility	
Provide patients, colleagues, and public officials with expert advice on preventing climate-related health risks	• In addition to patients, contacts may include those responsible for town planning and school education • Additional planetary health related counselling may not be feasible in all day clinical practice due to time constraints • Not specifically reimbursable
Be able to diagnose climate-related diseases	
Act as a multiplier of knowledge about planetary health	• A broad cross-section of society can be reached by medical doctors • Take advantage of the current public awareness of climate change
Act as a role model	• Doctors are often seen as trustworthy people
Motivate patients to take advantage of health-related benefits by living in a climate-friendly way	

*OSCE* objective structured clinical examination

**Table 2:** Feedback-related focus group interview with students who underwent the objective structured clinical examination on planetary health

What aspects of the feedback from the shadowers were meaningful to you?	
**Aspect**	**Additional comments**
Accompaniment through all OSCE stations	• Overall impressions of performance, especially of communication skills, are more reliable than impressions of individual OSCE stations • Communication skills improve from OSCE station to OSCE station (“learning by doing”) • Positive feedback increases self-confidence. Confidence in turn, strengthens the intention to address planetary health issues during a consultation • Some feedback was given between stations during the ongoing OSCE
Supplement to the examiner	• Shadowers’ assessment was mostly based on soft skills (relationship level) including body language and was therefore a good complement to the examiner’s assessment • Feedback was defused by the shadower (hardly critical) • Feedback on communication skills from shadowers was more differentiated than that from the examiners (where to develop?)
Independent observer	• The shadower may assess the OSCE performance differently from the examiner • Not all aspects of the content of an OSCE topic that the examiner expects the examinee to address may be evident from the situation. Effective communication of key messages as a proportion of the overall performance should therefore be more important than completeness • Feedback was independent of the final mark
How has the role change from examinee to shadower and vice versa affected your feedback behaviour?	
**Nature of change in feedback behavior**	**Additional comments**
Feedback remained largely unchanged by the impression of the shadower’s own OSCE	
Shadowers who have been through the OSCE have increased their understanding of candidates and examiners	• Understanding of incompleteness in favour of effectiveness in communicating key messages • Understanding of uncertain behaviour of candidates • Understanding of the assessment of examiners

*OSCE* objective structured clinical examination

### Statistical analysis

Categorical data are presented as counts and percentages. Student performance scores are presented as median and interquartile range. The Wilcoxon signed-rank test was used to compare paired samples. A 2-tailed value of *p* < 0.05 was considered as statistically significant. We performed the analysis using XLSTAT (version 2015.6.01.24026, Addinsoft, Paris, France).

## Results

### Participants

In the winter semester 2022/2023, a total of 20 students participated in the elective course on planetary health care and management. On 25 May 2023, a total of 12 students (60%) voluntarily completed the entire formative OSCE including all eight stations. A further eight students voluntarily completed four OSCE stations each, which included either the sub-themes of “physical activity”, “changing prescribing practice”, “nutrition”, and “vector-borne diseases” or the sub-themes of “mental health”, “management of heat waves in nursing homes”, “risk of preterm delivery due to heat stress”, and “use of specific inhalation anaesthetics”. The Likert-scale survey questions were responded by a minimum of 13 students and a maximum of 16 students each. A total of 13 (81%) students participated in the focus group interview.

### Student survey

Students agreed that most of the OSCE topics offered were likely to be useful in their future working life. Depending on the OSCE topic, between 60 and 100% of students agreed with the expected usefulness. The lowest level of agreement with usefulness was for the topics of “changing prescribing practice” (60%) and “mental health” (67%). Approximately one third of the students considered the OSCE topics of “change in prescribing practice”, “mental health”, and “collegial discussion about the type of inhalation anaesthesia” as difficult to accomplish.

Most students were in favour of integrating all the OSCE topics offered into compulsory courses. However, 21% of the students responded negatively to the topic of “changing prescribing practice” (Fig. 1).

### Student focus group interview

Students recommended that planetary health care and management should be included as a compulsory subject in medical education. As it requires a broad knowledge base, it should be taught in advanced semesters. This would also ensure that their basic knowledge is up to date when they start their careers. Students would prefer seminars or workshops to lectures because they allow them to actively contribute and train their communicative skills. In the students’ experience, learning success of OSCE was greater than that of workshops and online courses. Therefore, they suggest using OSCE as a regular additional teaching tool. Students described their role in relation to planetary health on several levels. Firstly, they feel obliged to behave in an environmentally friendly manner in their own area of agency, secondly, they want to be able to prevent, diagnose, and treat climate-related illnesses and to provide expert advice when needed, and thirdly, they want to motivate patients, staff, and colleagues to live and work in a climate-engaged manner and thus act as multipliers (Table 1).

Students appreciated the feedback from the shadowers. The shadowers were able to get an overall impression of the examinee’s performance as they accompanied the examinee through all the OSCE stations. Unlike the examiners, the shadowers focused more on the relationship level and communication skills and less on professional accuracy and completeness. The shadowers showed understanding for incompleteness in terms of intended content in favour of communicating key messages depending on the course of the conversation. They therefore complemented the examiner’s feedback in a meaningful way (Table 2).

Shadowers reported that they learned through observing of the OSCE and through specific reading inspired by the OSCE. Additionally, shadowing increased their attention to the time frame and structure of conversation situations (Table 3).

**Table 3:** Additional comments unrelated to questions during the focus group interview with students who underwent the objective structured clinical examination on planetary health

Additional comments regarding OSCE unrelated to the questions	
**Comment**	**Explanation**
Preparation of shadowers	• Shadowers reported that in the run-up to the OSCE they were not always sufficiently enabled (in time or properly) to assess the content-competence of the candidate, • Some shadowers were not aware that they were also required to give feedback on content-competence • Preliminary information in the form of numbered or bulleted lists was most appropriate
The shadower as learner	• Shadowers learned by observing the examinee’s performance and through specific reading up inspired by the shadowed OSCE
Shadowing can either improve or worsen one’s own OSCE performance	• Shadowing can give shadowers a knowledge advantage • Shadowing increases attention to time and structure • Shadowing can make shadowers insecure about their own performance as a candidate
Feedback on communication skills varied considerably between individual examiners	• In most cases, performance was judged to be good, but suggestions for further development were lacking • Examiners might have done better if they had more time
The establishment of fixed “triple teams” consisting of a candidate, an examiner, and shadower in all OSCE stations was proposed	• To improve the assessment of the candidate’s overall performance

*OSCE* objective structured clinical examination

### Student performance

**Fig. 2:**
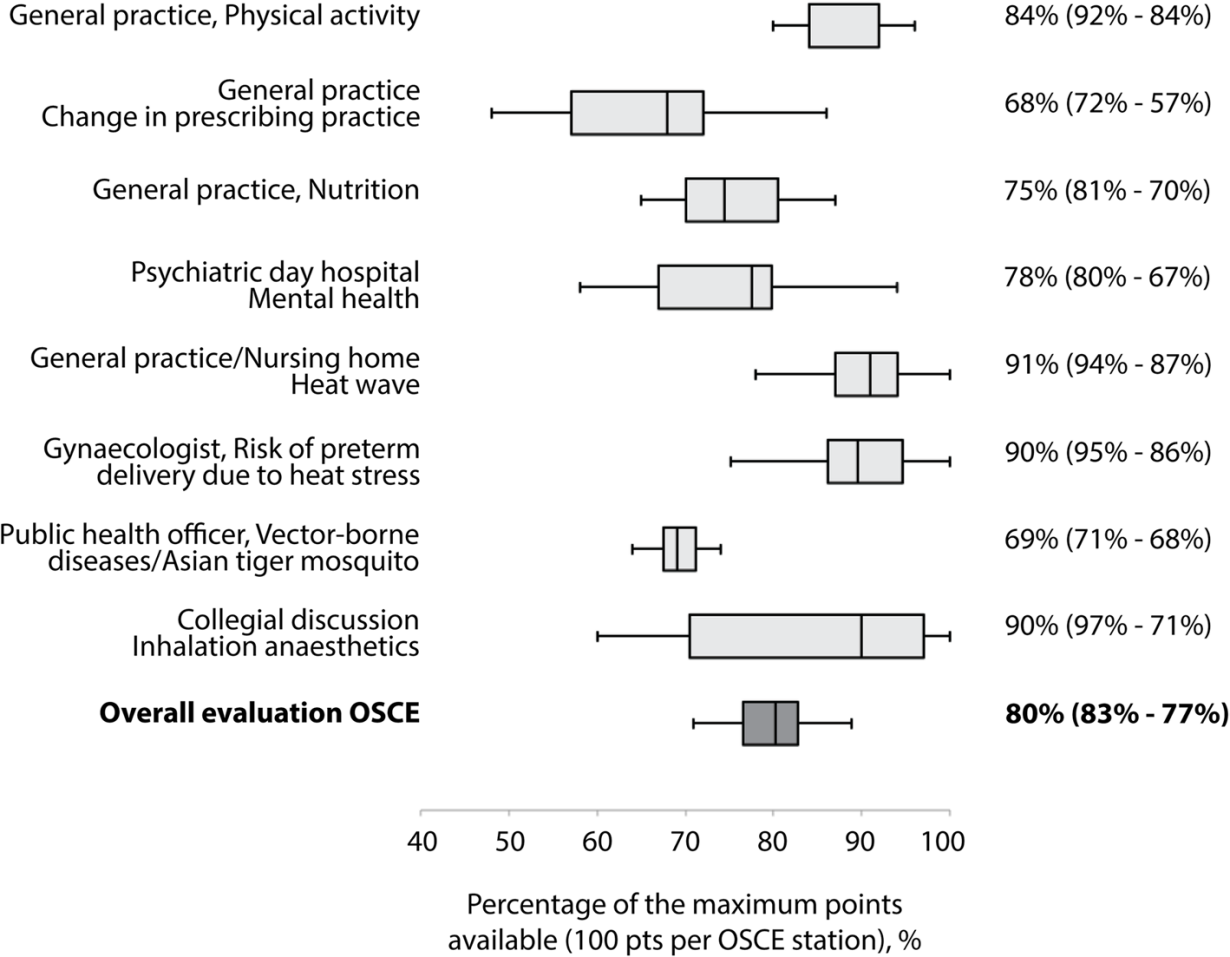
Assessment of students’ performance during the objective structured clinical examination (total examination: *n* = 12, individual stations: *n* = 16). Box plots show median and interquartile range and the lowest and highest data points within the 1.5 × interquartile range. OSCE, objective structured clinical examination

Examiners rated students’ performance ion the entire OSCE with a median of 80% of the maximum score (interquartile range [IQR] 83%—77%). The two lowest scoring tasks were advising an employee on changing prescribing practice (68%) and consulting a local mayor on community adaptation measures (69%). Performance on the task of collegial discussion of the type of inhalation anaesthetic used was rated with the largest IQR across all respondents (Fig. 2).

**Fig. 3:**
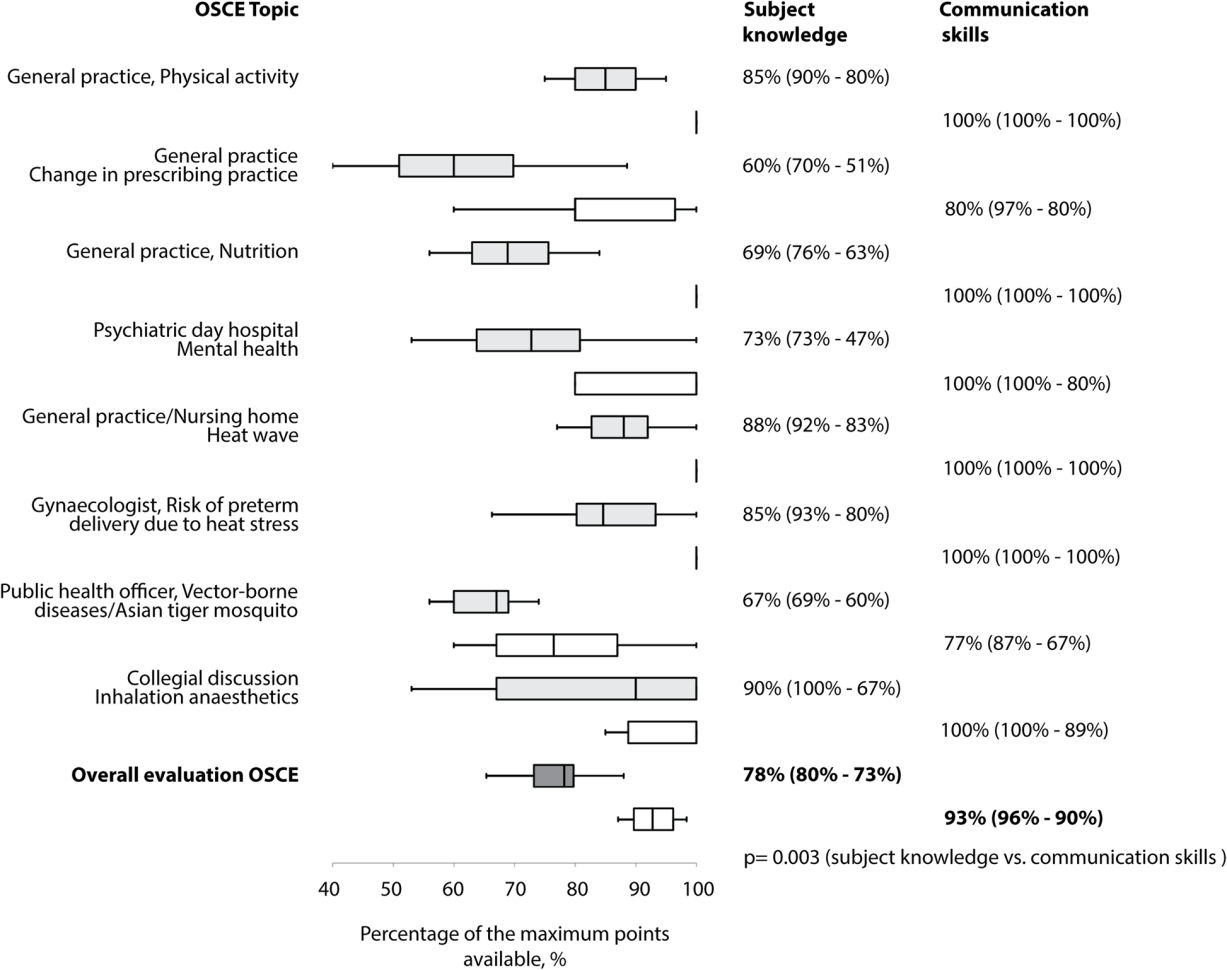
Assessment of students’ performance during the objective structured clinical examination distinguishing between subject knowledge and communication skills (total examination: *n* = 12, individual stations: *n* = 16). Box plots show median, interquartile range, and lowest and highest data point within the 1.5 × interquartile range. OSCE, objective structured clinical examination

Distinguishing between the assessment of subject knowledge and communication skills, examiners rated students’ performance in the overall OSCE as 78% (80%—73%) and 93% (96%—90%), respectively (*p* = 0.003). Students’ communication skills were rated most differently when the dialogue partner was an employee, a local authority or a colleague and the task was to initiate a transformation process (IQR 17%, 20% and 11%, respectively). Although communication skills in doctor-patient dialogue were rated with a median of 100% in all subtopics, there was considerable variation in the performance in giving medical advice on mental health (IQR 20%) (Fig. 3).

## Discussion

This observational study evaluated the suitability of OSCE to train skills needed for planetary health care and management. Advanced semester students voluntarily underwent OSCE after participating in self-directed online courses and workshops. A subsequent survey and group interview revealed that students supported the inclusion of planetary health topics in the form of required seminars or workshops into the advanced medical curriculum. The OSCE as a teaching tool was seen as an efficacious complement. Additional learning was facilitated by shadowing peers during the OSCE with the objective to provide feedback.

Healthcare professionals have a high level of credibility with the public. They are therefore well suited to raise awareness and motivate patients to make climate-friendly lifestyle changes that benefit both the planet and the patient. However, planetary health care and management poses specific communication and argumentation challenges for physicians. It is useful to emphasise opportunities and benefits that directly affect people, such as health co-benefits, and to avoid negative warning about dangers, burdens, and losses [14]. The rhetoric of intractability contributes to instill paralysis. Instead, problem descriptions should be matched with feasible solutions and intervention perspectives [15]. To deal with feelings of doom and powerlessness, stories of steps to be taken are conductive [16]. Communication skills should therefore be taught and practiced alongside expertise on planetary health.

Our study showed that students’ communication skills were lowest in situations with employees, officials, or colleagues. However, such challenging conversational scenarios will undoubtedly occur in professional life as adaptive transformations of existing practices and structures need to be encouraged by health care professionals [17]. In this respect, leadership development and addressing of team dynamics within the medical curriculum as well as management practice at the latest during working life may improve confidence and skills [18–20]. In addition, students could be involved in collaborations through their university as an academic partner of local authorities. However, an increase in expert knowledge could also improve the conversation in the classic doctor-patient dialogue. This could be particularly true in the area of mental health.

Previous research has shown that formative assessment with immediate feedback during the learning phase improves clinical competence [21]. OSCE training was found to be effective in improving counselling and communication skills and self-confidence [12]. Students found OSCE to be enjoyable and a welcome change from routine [11]. Students who expected to be assessed by OSCE rather than multiple-choice questionnaires focused more on acquiring clinical skills in authentic learning environments and needed less study time to achieve the expected level of competence [22]. OSCE used as a learning tool for interpersonal and communication skills in challenging conversations allows students to practice active listening and discussion. Feedback from examiners and shadowers facilitates personal and guided reflection, i.e., the cognitive and affective exploration of the experience over time. Experience and reflection are intended to develop and refine individual competence at skill-level. In addition, shadowers benefit from observational learning from the peer’s actions and from the examiner’s reinforcing responses to the peer [13, 23].

In our study, from the students’ point of view, efficacy of feedback from examiners is worthy of improvement. The examiners probably assumed a higher level of competence in interpersonal and communication skills than was actually the case and did not assess these competences in a sufficiently differentiated way. The feedback was not very constructive. Therefore, assessors should be made aware to consider that feedback should be specific, balanced, and constructive. It should describe behaviours and gaps in students’ learning [24]. Feedback is not a one-way statement but rather a conversation [25]. Previous studies confirmed our finding that peers can provide valuable feedback on OSCE performance [23, 25–27].

A possible disadvantage of OSCE is, however, that it requires considerable resources and amount of time for thorough preparation, implementation, and administration [9, 11]. On the other hand, the proportion of self-directed learning using online platforms could be increased, and lectures reduced. The positive assessment of the OSCE performance in our study shows that a combination of self-directed learning and subsequent workshops is essentially adequate to achieve a sufficient learning success. Trained advanced students (peers or near-peers) could be involved in the design and revision of OSCE stations and replace experienced clinical teachers as peer assessors. in this way they can consolidate their knowledge, develop teaching skills, and experience a sense of leadership [11, 25, 28].

There are some limitations to our research. First, participation in the online learning platform, workshops and the OSCE on planetary health was voluntary. Therefore, volunteers were particularly motivated to learn on their own initiative and may be biased. Secondly, the total number of students who participated in the study was small. Both of these issues limit the generalizability of our findings. Whether planetary health can be included in the medical curriculum as a required or elective course may depend on the specific circumstances of each university. In our case, we have integrated planetary health into our reformed “JENOS” programme, which covers 15% of the clinical curriculum and allows students to choose between different tracks (clinical, outpatient, or research-oriented medicine) according to their personal interests [29]. In either case, however, the OSCE can be used as an effective teaching tool.

## Conclusions

Students rated their learning from the final OSCE through experience, observation, and feedback as higher than their learning from the preparatory online courses and workshops. OSCE requires the combined application of knowledge and communication skills necessary to raise awareness and empower dialog partners to achieve both health and environmental objectives. Examiners’ evaluation of the students’ OSCE performance revealed weaknesses, particularly in the dialogue with employees, colleagues, and an administrative officer, which can be addressed in future training. Students appreciated the giving and receiving of peer feedback on their OSCE performance. Finally, they recommended the inclusion of planetary health as required courses in the advanced curriculum. OSCE used as a complementary teaching tool, can further enhance professional competence in planetary health care and management.

### Supplementary Information

**Supplementary Material 1. **

## Data Availability

No datasets were generated or analysed during the current study.

## Abbreviations

OSCE: Objective structured clinical examination
MDI: Metered dose inhaler
