# Supplementary material for: Objective structured clinical examination to teach competency in planetary health care and management – a prospective observational study
Source: BMC Med Educ. 2024 Mar 19;24:308. doi: 10.1186/s12909-024-05274-9 (PMC10953132; doi:10.1186/s12909-024-05274-9)
Supplement: Supplementary file 1 — Supplementary Material 1. [file 12909_2024_5274_MOESM1_ESM.docx]

**Supplemental Table 1** Proportion of the total score as a measure of student performance in each OSCE station

| **OSCE station** | **Anamnesis** | **Environ-mental impact / health co-benefits** | **Option for action** | **Communi-cation skills** |
| --- | --- | --- | --- | --- |
| Advice on physical activity to an overweight patient. | 20% | 40% | 20% | 20% |
| Discussion with an employee about changing medical prescribing practice. | 0% | 30% | 40% | 30% |
| Dietary advice to an overweight patient. | 20% | 45% | 15% | 20% |
| Environmental risk assessment for mental health of a depressed patient. | 25% | 30% | 25% | 20% |
| Advice to a nursing care expert on measures to be taken during heat waves. | 0% | 20% | 55% | 25% |
| Medical advice to a pregnant woman on the risk of preterm delivery due to heat stress. | 0% | 52% | 24% | 24% |
| Consultation with a local mayor on the Asian tiger mosquito. | 0% | 40% | 30% | 30% |
| Collegial discussion on inhalation anaesthetics. | 0% | 60% | 0% | 40% |
